# Conservative treatment of fingertip injuries in children – first experiences with a novel silicone finger cap that enables woundfluid analysis

**DOI:** 10.3205/iprs000125

**Published:** 2018-10-19

**Authors:** Jurek Schultz, Percy Schröttner, Susann Leupold, Adrian Dragu, Silvana Sußmann, Michael Haase, Guido Fitze

**Affiliations:** 1 Pediatric Surgery, Faculty of Medicine Carl Gustav Carus, TU Dresden, Dresden, Germany; 2 Institute of Medical Microbiology and Hygiene, Faculty of Medicine Carl Gustav Carus, TU Dresden, Dresden, Germany; 3 Department of Plastic and Hand Surgery, Faculty of Medicine Carl Gustav Carus, TU Dresden, Dresden, Germany

**Keywords:** fingertip regeneration, fingertip amputation, fingertip injury, infection, wound fluid, pediatric surgery, occlusive dressings

## Abstract

**Introduction:** Human fingertips are able to regenerate soft tissue and skin after amputation injuries with excellent cosmetic and functional results when treated with semiocclusive dressings. Despite bacterial colonizations, proceeding infections are not reported with this management. The underlying mechanisms for this form of regenerative healing as well as for the resilience to infections are not known. Due to the lack of mechanical protection, the leakage of maloderous woundfluid and the sometimes challenging application, conventional film dressings have their problems, especially in treating young children. We therefore treated selected patients with a novel silicone finger cap with an integrated wound fluid reservoir that enables atraumatic routine wound fluid aspiration.

**Methods:** We report on 34 patients in between 1 and 13 years with traumatic fingertip amputations primarily treated with occlusive dressings. 12 patients were treated with a novel silicone finger cap. We summarized clinical data for each patient. This included photographs and microbiological results from wound fluid analyses, whenever available.

**Results:** The results of both, conventional film dressing and silicone finger cap treatment, were excellent with no hypersensitivity and no restrictions in sensibility and motility. Even larger pulp defects were rearranged in a round shape and good soft tissue coverage of the distal phalanx was achieved. Nail deformities were not observed. We detected a wide spectrum of both aerobic and anaerobic bacteria in the wound fluids but infections were not observed. Epithelialization times did not differ significantly and no severe complications were seen in all primarily conservatively treated patients.

**Conclusion:** This study provides preliminary data demonstrating that the treatment with the silicone finger cap leads to excellent clinical results in wound healing. Interestingly, the wounds were colonized with a wide range of bacteria including species that may cause wound infections. However, we saw no proceeding inflammation and the regeneration was undisturbed. In the future, the efficacy of this new management should be evaluated in randomized, controlled clinical trials to confirm the results under standard conditions and get more insight into the role of the wound microbiome as well as other factors that may promote regeneration. The aspirable Reservoir of the finger cap will enable easy atraumatic sampling of wound fluids both for diagnostic and for research purposes as well as possibly allowing direct administration of pro-regenerative drugs in the future.

## Zusammenfassung

**Einleitung:** Fingerendglieder können nach Amputationsverletzungen mit hervorragenden kosmetischen und funktionellen Ergebnissen regenerieren, wenn die Verletzungen mit semiokklusiven Verbänden behandelt werden. Trotz der bakteriellen Kolonisation dieser Wunden werden keine fortschreitenden Infektionen berichtet. Der Mechanismus für diese regenerative Heilung und die Resilienz gegen Infektionen ist unbekannt. Aufgrund der Leckage von übelriechender Wundflüssigkeit und wegen des Fehlens eines mechanischen Schutzes sowie durch die mitunter schwierige Anlage selbstklebender Folien, sind diese bei der Versorgung von Kindern teilweise problematisch. Daher behandelten wir ausgewählte Patienten mit individuell gefertigten Silikonfingerlingen. Dabei wurde regelmäßig Wundflüssigkeit aus einem integrierten Reservoir aspiriert und bakteriologisch untersucht.

**Material und Methoden:** Wir berichten über 34 Patienten zwischen 1 und 13 Jahren mit traumatischen Fingerendgliedteilamputationen, welche primär semiokklusiv behandelt wurden. 12 Patienten wurden dabei mit dem neuartigen Fingerling behandelt. Wir werteten retrospektiv die Patientenakten aus. Dies beinhaltet Fotodokumentationen und mikrobiologische Untersuchungsergebnisse wenn vorhanden.

**Ergebnisse:** Die Ergebnisse beider Behandlungsverfahren waren sehr gut: Es gab weder Sensibilitätsstörungen noch Bewegungseinschränkungen. Auch nach größeren Weichteildefekten resultierten kosmetisch schöne Fingerkuppen mit guter Weichteildeckung der distalen Phalanx. Nageldeformitäten wurden nicht festgestellt. In den bakteriologischen Untersuchungen der Wundflüssigkeiten fand sich ein großes Spektrum an aeroben und anaeroben Keimen. Fortschreitende Infektionen gab es nicht. Die Heilungszeiten waren vergleichbar bei beiden Behandlungsformen. In den primär konservativ behandelten Patienten wurden keine ernsten Komplikationen festgestellt.

**Conclusio:** Fingerendgliedteilamputationen bei Kindern können grundsätzlich erfolgreich konservativ mit dem neuen Silikonfingerling behandelt werden. Die Wunden sind dabei mit Bakterien kolonisiert. Dabei sind auch als Erreger von Wundinfektionen bekannte Keime. Dennoch kam es nicht zu fortschreitenden Infektionen und die regenerative Heilung schien nicht gestört. In Zukunft bedarf es randomisierter, kontrollierter Studien, um die Wirksamkeit des Silikonfingerlings nachzuweisen und mehr über die Rolle des Mikrobioms und anderer Faktoren zu erfahren, die möglicherweise die Regeneration beeinflussen. Durch das punktierbare Wundflüssigkeitsreservoir im Silikonfingerling kann dabei Wundflüssigkeit regelmäßig für diagnostische und wissenschaftliche Zwecke entnommen werden. Weiterhin könnten in Zukunft regenerationsfördernde Substanzen auf diesem Weg atraumatisch in die Wunde eingebracht werden.

## Introduction

In pediatric emergency departments, fingertip injuries account for up to 2% [1] of presentations of children under 14 years of age with 25% of these injuries being more serious and needing surgical treatment, in 15% of the cases under general anesthetics [1]. Already in 1974, Illingworth reported on pediatric fingertip injuries with substance loss, who were treated conservatively with very good success [2]. The superiority of the conservative approach in comparison to surgical managements was demonstrated thereafter [2], [3], [4], [5]. Later, sulphadiazine gloves and thus a method of occlusive dressing that formed a wet chamber around the injury, were used [6]. Söderberg et. al. used ink tattoos on the wound edge to show that human fingertips regenerate de novo under conservative treatment [7]. Additionally, the use of film dressings to occlude the injured fingertips was demonstrated in a series of 200 patients in 1993 [8]. For the last 25 years, authors have concluded that this kind of treatment should be recommended for all fingertip injuries [8], [9].

Surgical approaches for fingertip amputation injuries that cannot be subjected to primary closure include stump plasties, local or distant flaps [10], microsurgical replantation, composite grafts or skin transplants [11]. There is only little and conflicting data on the indication of conservative and surgical techniques. Controversies also exist on necessary wound disinfection, if exposed bone should be shortened before any occlusive dressing is applied and, whether or not, amputates should be reattached as composite grafts [11], [12].

The ability to regenerate severed limbs is well known in nature. The most astonishing results can be found in amphibians [13]. In mammals, this regenerative capacity is reduced during ontogeny. In grown up humans, limb regeneration can only be displayed at the distal fingertip. The underlying mechanisms as well as an explanation why limb regeneration in humans is confined to the fingertip remain unknown. 

Cellular and soluble factors play a role in the regeneration of injured fingertips [14], [15]. Their pro-regenerative effects seem to be promoted in a wet chamber as it is provided by an occlusive dressing. Furthermore, under occlusive environments there is enhanced cell migration and re-epithelialization [16]. The rate of infections can be markedly reduced under occlusive dressings [17] and finally there is reduced inflammatory response possibly due to the maintenance of normal cellular hydration [18], [19]. Therefore occlusive dressings of injured fingertips follow the modern principles of wound care.

In many centers the conservative management of fingertip injuries is accomplished with self-adhesive film-dressings [8], [20]. However, this management is sometimes difficult, especially since these dressings do not stick to wet skin. Conventional dressings do not form a protected chamber around the wound, additional splinting is often needed [5], [6]. The leakage of malodorous wound fluid is very disturbing and in some cases wounds can even dry out completely leading to suboptimal results [21]. Therefore we optimized the conventional occlusive dressings by applying techniques known from silicone finger ortheses and designed a novel silicone finger cap that deals with the aforementioned issues. 

Thus far it has been very difficult to analyze wound fluid from human fingertip injuries during regeneration [14], [15]. The reservoir at the tip of our silicone finger cap allows aspirating excess wound fluid without disturbing the healing process and without causing pain. Therefore it is possible to routinely gain samples of wound fluid from regenerating human fingertips for clinical diagnostics and research. 

## Objective

In order to gain insight into different means of occlusive therapy for traumatic partial fingertip amputations used in our clinic and to evaluate our first experiences with silicone finger caps, we report on 34 pediatric patients treated in between 2012 and 2013 primarily with convential occlusive dressings (n=22) or our silicone finger cap (n=12). We designed and constantly improved this finger cap to provide a more pleasant therapy for our patients that possibly also provides a better environment for the regenerating fingertips. We also retrospectively evaluated our microbiological routine diagnostic results to gain information on the microorganisms present.

## Materials and methods

### Inclusion criteria

Our inclusion criteria were all full skin substance defects distal to the distal interphalangeal joint (DIP-joint) in between 1^st^ January, 2012 and 31^st^ December, 2013 unsuitable for primary surgical closure without further substance loss. Injuries with exposed bone as well as injuries to the bony distal phalanx itself were included. The application of the finger cap had to take place within 48 hours after the trauma. In between the injury and the application of the finger cap, the fingers were occluded with conventional film dressings. Secondary occlusions after more than 48 hours, animal bite injuries [22] or patients who had surgical interventions were excluded from this retrospective evaluation. This study has ethical board approval (EK116032016).

### The silicone finger cap

**Figure 1:**
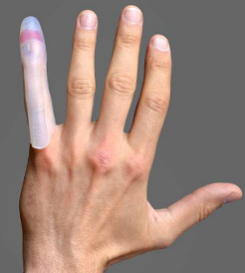
Photograph of silicone finger cap

**Figure 2:**
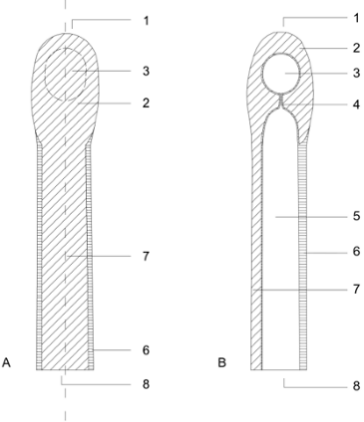
A) Dorsal view of the silicone finger cap, B) Cross section of silicone finger cap 1=apex, 2=reinforced tip, 3=reservoir for woundfluid, 4=capillary connection to finger chamber, 5=chamber modelling the original shape of the finger, 6=thin and very soft wall of the finger cap, 7=reinforced dorsal bridge, 8=base

The silicone finger cap consists of a thin and soft shaft that surrounds the base of the finger and provides the semi-occlusive seal without the need for additional adhesives (Figure 1 (Fig. 1)). It forms a protected chamber of more rigid silicone around the distal phalanx that is close to the original, anatomical form and size of the fingertip. The more rigid silicone is continued in a narrow bar all the way to the base of the finger cap thus splinting the injured finger enough to care for undisturbed healing while allowing some movement in all finger joints. A reservoir for excess wound fluid is connected to the wound chamber by capillaries, thus enabling free diffusion in between the wound and the reservoir (Figure 1 (Fig. 1), Figure 2 (Fig. 2)). This reservoir can be punctured with a regular injection needle. The used medical silicone (*Dragon Skin Series Part A&B* from the company KauPo, *Silastic*® *Q7–4720 Biomedical Grade ETR Elastomer* and *Silastic*® *Q7–4765 Biomedical Grade ETR Elastomer* both from the company Dow Corning) is permeable to oxygen to some extent but impermeable to water vapour. For this series all finger caps were individually handcrafted by Orthopedic and Rehabilitation Engineering Dresden (ORD), Dresden, Germany. 

### Treatment algorithm

Haemostasis was achieved by elevation and gentle pressure. 22 reviewed injuries werde occluded with conventional film dressings (Suprasorb F, Lohmann & Rauscher, Germany). These patients were treated according to different protocols with or without disinfection of the wound. Patients with film-dressings usually received an additional protective finger splint. Treatment algorithms for conventional film dressings were not standardized and often they were not recorded in the patients notes in detail. In general, patients were reviewed weekly to change the outer gauze dressings. Every two weeks, the film dressing was removed to assess epithelialization. However, unscheduled dressing changes were performed due to the leakage of maloderous wound fluid.

Treatment with the finger cap always followed the same protocol, initiated at latest within two days after the injury. Parents or patients were offered the treatment with a silicone finger cap if the wound was deeper, i.e. Type II or III according to the Allen classification [23] or if the patient or parents asked for a more convenient therapy. Wounds were thoroughly cleaned with isotonic saline solution. Disinfecting agents were not used. Antibiotics were not administered. Using a set of finger cap dummies the correct diameter is selected on the corresponding finger of the contraleteral hand. The finger cap can be cut to length using conventional scissors. The transparent material of latest generation finger caps makes it easy to verify the correct fitting. We conducted a first clinical control, aspiration of wound fluid and renewal of outer gauze dressings within 24 hours of the initial application to ensure excellent fitting of the finger cap. Thereafter we conducted clinical controls, aspiration of wound fluid and renewal of outer gauze dressings weekly. Biweekly we additionally removed the finger cap to photograph the injury and document regeneration progress. If epithelialization was close to complete, we sometimes planned for the removal already one week later. Whenever sufficient wound fluid could be aspirated, swabs were sent in transport medium (Sterile Transport Swab, NUOVA APTACA SRL, Canelli, Italy) for microbiological cultures and Gram-staining. Final assessment and documentation of the clinical outcome was done on 7 of 9 evaluated cases (two were lost to follow-up) in between 1.3 to 7.5 months after the injury.

### Microbiological analysis

Gram-staining was carried out and evaluated. Smears were plated on Colombia Blood Agar containing 5% sheep blood, Bile-Chryosidin-Glycerol agar (BCG-Agar) for selection of Gram-negative, Sabouraud agar for identification of yeasts and Glucose Yeast Extract Cysteine agar for isolation of anaerobic bacteria. Aerobic bacterial growth was checked after 24 and 48 hours. Anaerobic cultures were evaluated after 48 hours and 7 days. The identification of bacterial species was performed by automated systems VITEK 2 (bioMérieux, Nürthingen, Germany) and/or MALDI-TOF MS (Bruker Daltonics GmbH, Bremen, Germany). Antimicrobial resistance profiles were performed by VITEK 2 (bioMérieux, Nürthingen, Germany) and/or E-test stripes (bestbion dx GmbH, Cologne, Germany). E-Tests were performed and MIC values evaluated according to EUCAST (“The European Committee on Antimicrobial Susceptibility Testing”. Breakpoint tables for interpretation of MICs and zone diameters. Version 5.0, 2015. http://www.eucast.org). Additionally, of 22 patients treated initially with conventional semi-cocclusive film dressings, microbiological analyses were carried out in 9 visits of 4 patients when dressings had been completely removed. 

### Data 

We collected all data written in the patients charts and in our computer system during the treatment process. Additionally we used the photographs from the injured fingers and the microbiological analyses.

## Results

### Descriptive analysis of patients and injuries

**Figure 3:**
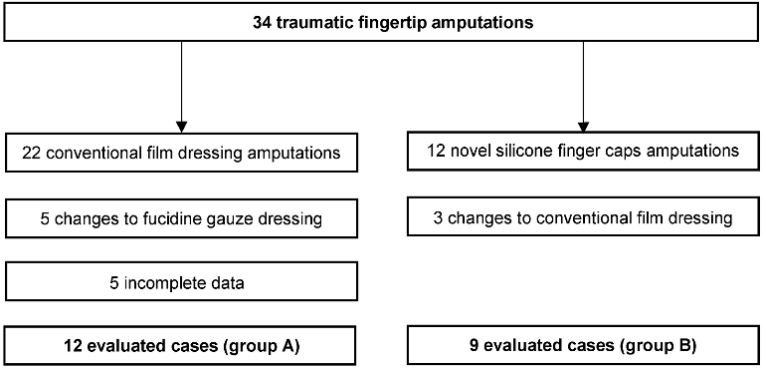
Overview of patients included in this study

In between 2012 and 2013, we conservatively treated 34 patients with traumatic partial amputation injuries, of whom 22 were treated with conventional occlusive film dressings (group A) and 12 with our novel silicone finger cap (group B) (Figure 3 (Fig. 3)). In group A, the index finger was most frequently affected and the majority of the injuries were cutting injuries (Table 1 (Tab. 1)). Taken together, we treated 12 girls and 10 boys in between 1 and 13 years (mean age 7.0 years) with a convential occlusive dressing. No fractures were seen in this group. Injuries treated conventionally were not rated according to any established classification. We calculated clinical results for 12 patients since five patients were changed to fucidine gauze dressings due to minor complications or patient complains regarding the disturbing odour. Another 5 patients were excluded because they were lost to follow-up or available data was incomplete.

During the same time, we used our novel silicone finger cap on 12 patients with traumatic partial amputation injuries to the fingertip. Primarily operated injuries were excluded. One bite injury is not included in this series but reportet separately [22]. 5 type I injuries, four type II injuries and three type III injuries according to Allen 1980 [18] were treated. Index and ring fingers were most frequently affected (Table 1 (Tab. 1)). In seven cases the nail bed was affected. Of these seven cases, three had an injured or partially amputated lunula. In 4 cases an X-ray showed a tuft fracture. In total, we report on 4 boys and 8 girls in between 1 and 12.8 years of age. The mean age was 7.7 years.

The most common accident was a cutting accident with an automatic bread slicer (n=4). In 2 cases the injury was caused with a knife and one finger was cut with a shard of glass. Five patients had jammed their finger either in a closing door (n=3) or in between a scooter and a wall or underneath a foot. We reported our clinical results for 9 finger cap occlusions, since three patients were changed to other treatments (Figure 3 (Fig. 3)).

### Clinical outcome

**Figure 4:**
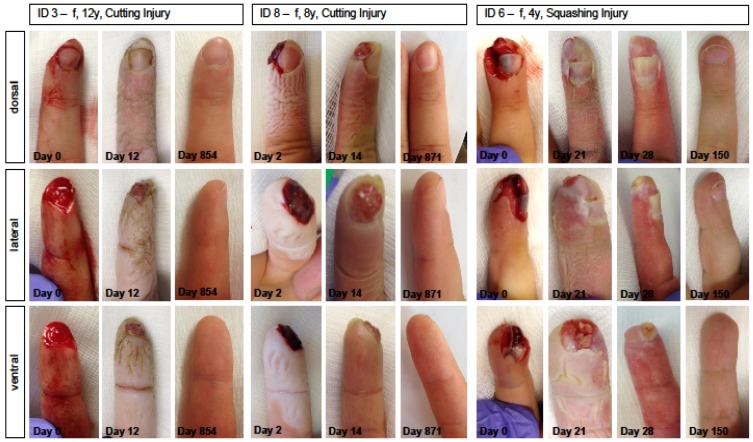
Photo documentation during clinical course and outcome at follow-up of three exemplary patients treated with silicone finger cap In two cases there was a minimal residual non-adherence of the nail body to the distal nail bed. Only in two cases we saw an interruption of the regularly regenerated pattern of dermal ridges by a small scar. Epithelialization time reached from 20 to 36 days in the patients treated with a finger cap. Where data was available, film occlusions were found to be equally successful with epithlialization times from 6 days to 36 days (Table 2).

The results at the follow-up visit (1.3 to 7.5 months after the trauma) were excellent: We saw no restrictions in sensibility and motility. Hypersensitivity was not reported. Even larger pulp defects were rearranged in a round shape and normal soft tissue coverage of the distal phalanx was achieved. Common nail deformities like parrot beak or relief inconsistencies were not observed (Figure 4 (Fig. 4)).

In two cases there was a minimal residual non-adherence of the nail body to the distal nail bed. Only in two cases we saw an interruption of the regularly regenerated pattern of dermal ridges by a small scar. Epithelialization time reached from 20 to 36 days in the patients treated with a finger cap. Where data was available, film occlusions were found to be equally successful with epithelialization times from 6 days to 36 days (Table 2 (Tab. 2)).

### Complications

Out of 12 patients treated with the finger cap, one patient was changed after one day to a film occlusion because the initial finger cap was measured too small. Additionally, two toddlers who were treated with an early design finger cap with a very thick wall in the basal shaft area were changed to a film dressing due to skin irritations at the bases of the finger cap (Figure 3 (Fig. 3)). Furthermore, we had to perform one additional, unplanned dressing change because of sub-febrile temperatures with unknown focus. We prescribed 3 days of oral cefuroxime even though a local inflammation was not seen (Table 3 (Tab. 3), patient ID 7). The further course of the treatment of all patients mentioned above was successful and the final outcomes were excellent.

Among the patients treated initially with a film dressing, five patients were changed to fucidine gauze dressings because parents did not want to tolerate leakage of wound fluid and disturbing odours. One patient generated a rather large hypergranulation under the film dressing that was successfully treated with silver-nitrate.

### Microbiology

Besides normal aerobic and anaerobic skin flora, the bacteria in wound fluid aspirates from finger caps identified to genus or species level are summarized in Table 3 (Tab. 3) and Table 4 (Tab. 4). Similar bacterial species were seen in all patients regardless whether they were treated with finger caps or conventional semi-occlusive techniques. However, besides skin flora, no Gram-negative bacteria were identified in patients treated with film dressings.

## Discussion

Conventional film dressings for the semi-occlusive treatment of fingertip injuries are sometimes challenging to apply, especially on bleeding wounds and in pediatric patients. After the application of film dressings, we have often felt the need for additional splinting of the injured finger for mechanical protection of the wound. Furthermore, many patients and parents found constant leakage of malodorous wound fluid very disturbing, especially in a sometimes unforgiving social environment such as kindergarten or school. In summary of these problems, some authors even concluded, that the treatment of fingertip injuries by occlusive devises is not suitable for children [5], [6]. To our knowledge, there is currently no suitable occlusive dressing available, that is classified as type IIb medical device and certified for longterm use on deep wounds.

We therefore designed the described silicone finger cap. There is no need for excessive manipulation of the finger, especially no drying is needed, since the finger cap does not depend on any adhesives. Excess wound fluid is collected in a reservoir at the tip of the finger cap thus giving additional protection to the wound surface as well as minimizing leakage of wound fluid at the base of the finger cap. A bridge of more rigid silicone is incorporated in the finger cap. This bridge is splinting the injured finger and protects the chamber around the wound to allow for undisturbed regeneration. For clinical monitoring, our finger cap is made of transparent and radio-translucent silicone. 

The reservoir for excess wound fluid also allows sampling of the wound fluid that surrounds the fingertip injury during regeneration without disturbing the regeneration process or causing discomfort or pain. By the same route, medications to foster the regeneration process could be administered directly into the wound fluid in the future.

Three complications were encountered in the finger cap group: in one case we felt the initial finger cap was a little small in diameter and therefore changed to a film occlusion. Two further patients showed skin irritations at the base of the finger cap due to early thick-walled finger caps rubbing against the volar skin when the MCP-joint was in maximal flexion. Both problems could be solved for future patients technically: There is now a simple way of finding the correct diameter by employing a set of measuring rings on the corresponding finger of the contralateral hand. Furthermore the ventral wall of the finger cap has been changed to be fabricated as thin as a conventional condom. Therefore the finger cap is less likely to excert any kind of compression and basal skin irritation has not been a problem ever since.

In the conventional film dressing occlusions (group A) we saw a comparable rate of patients that had to be changed to different therapies (5 out of 22 vs 3 out of 12). The clinical results were comparable in both groups. Epithelialization times are difficult to compare due to the variety of different wounds with inconclusive classifications and highly individual injury mechanisms. So far, we cannot state that any means of occlusion is leading to faster epithelialization. 

### Microbiology 

The overall risk for infections in conservatively treated fingertip injuries is very low. According experiences gained from semi-occlusively treated wounds, the infection rates under occlusion are even lower than under conventional gauze dressings [17]. In several large series of semi-occlusively treated fingertip injuries, there is no report of a clinically relevant infection [3], [8], [20], [24].

The necessity, mode and extend of wound disinfection prior to the occlusion of fingertip injuries is reported controversially. Many authors confine themselves to cleansing of the wound with sterile sodium solution without encountering any clinically relevant infections [2], [5], [7], [25]. While not reporting on treatment-relevant infections either, other authors, who employ rigorous disinfection protocols, find regular colonization of the wounds with a variety of bacteria similar to the spectrum of organisms we report in this series [3], [8], [15].

Some authors see the commensal skin flora as a protection against clinically relevant infections with pathogens and consequently omit wound disinfection prior to occlusion [26]. Following this line of arguments and in order to limit painful manipulation on our pediatric patients to an absolute minimum, we did not use wound disinfections. Due to this protocol, we have not seen the need for painful finger block anesthesia.

Local signs of inflammation or infection were absent in all cases we observed in this report. This is of special interest, since some bacteria we identified can indeed cause wound infections after trauma or surgery. The skin-dwelling bacterium *Staphylococcus aureus* for instance, inherits a large number of different virulence factors (such as collagenases, lipases and hyaluronidases) which enable invasive wound infections, including abscesses and cellulitis that may also lead to blood stream infections (Table 3 (Tab. 3) and Table 4 (Tab. 4)).

Further bacteria associated with wound infections in immunocompromised patients or after trauma respectively surgery, were identified in our specimen: *Acinetobacter pitii* [27], [28], [29] (a Gram-negative non-fermenting rod of growing clinical importance), *Acinetobacter ursingii* [30] (a species related to *Acinetobacter baumannii*), *Leclercia adecarboxylata* [31], *Citrobacter brakii* [32] (both are Gram-negative rods belonging to the family of Enterocerobacteriacea), *Bacillus cereus* [33] and *Bacillus pumilus* [34], Gram-positive spore-forming rods, which are ubiquitously found in the environment and mostly regarded as contaminants. *Brevibacillus parabrevis* was detected in one case (patient ID 1). It is a Gram-positive, motile aerobic rod with a still unresolved role in human pathogenecity. 

In addition, wound infections caused by *Fusobacterium* spp. (Gram-negative anaerobic, non-spore forming bacteria, which are part of human oral flora and primarily cause peridontal diseases), *Neisseria elongata or Eikanella corrodens* [35], [36] (a fastidious, Gram-negative bacterium, which belongs to the family of Neisseriaceae and is also part of the normal mucosal flora) that are seen after bite injuries could be identified in our study [37], [38]. 

We also identified *Corynebacterium amycolatum*. It has been shown to be a causative agent for prosthetic joint, blood stream infections and endocarditis [39], [40], [41]. Since it predominantly causes infections in immunocompromised patients it is seen as an emerging pathogen [42].

Moreover, we found members of the genus Clostridium. However, we could not define the species. These bacteria are strictly anaerobic and ubiquitously found in nature. The species *Clostridium perfringens, septicum, novyi* and *histolyticum* cause one of the most worrisome diseases, the trauma-associated gas gangrene. This disease warrants an immediate surgical intervention and is associated with fatal outcome.

Despite of a severe trauma and the aforementioned colonializations, we could not find proceeding wound infections. This may be explained by some fundamental mechanisms. On the one hand, bacterial growth could be inhibited by the innate immune system. Zhang et al. for example demonstrated that antimicrobial peptides produced by adipocytes are able to eliminate *Staphylococcus aureus* and thus inhibit skin infections [43]. Moreover, many bacteria competing for the same ecological alcove may restrict the growth of pathogenic bacteria. 

*Lactobacillus fermentum* for example is able to limit the growth of *Staphylococcus aureus* [44]. Similarly microcins may eliminate Enterobacteriaceae. Microcins are small bacterial molecules which help to inhibit the growth of rival species [45]. Future research will investigate the microcine production of bacteria under semi-occlusive conditions and possibly even elicit a beneficial role of certain bacterial colonialization to fingertip regeneration.

## Conclusions

Fingertip injuries in children have an astonishing potential for regenerating ad integrum under semi-occlusive conditions. Despite being colonized by a variety of different bacteria, they can heal without infectious complications. The role of bacteria identified in wound fluid during fingertip regeneration will be further examined in future research [46]. Our silicone finger cap provides a save, comfortable and easy to handle device to establish a protected semi-occlusive dressing around injured fingertips that overcomes many problems of conventional film dressings. The finger cap also permits routine aspiration of wound fluid for diagnostic and research purposes thus facilitating future research into soluble factors that foster regeneration of human fingertips. Our finger cap also allows for painless administration of medications that prospectively could promote fingertip regeneration.

## Notes

### Statement of human rights

All procedures performed in studies involving human participants were in accordance with the ethical standards of the institutional and/or national research committee and with the 1964 Helsinki declaration and its later amendments or comparable ethical standards. This study has been approved by the responsible Ethic Committee (approval no.: EK 116032016).

### Conflict of interest

The TU-Dresden, Dr. Jurek Schultz and Prof. Guido Fitze have filed a patent application for the silicone finger cap (PCT/DE2014/100088; 14721743.4–1308; 14/774,997). Besides, the authors declared no potential conflicts of interest with respect to the research, authorship, and/or publication of this study.

## Figures and Tables

**Table 1:**
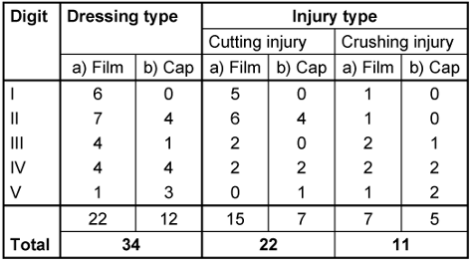
Distribution of injuries to digit number and mechanism of injury

**Table 2:**
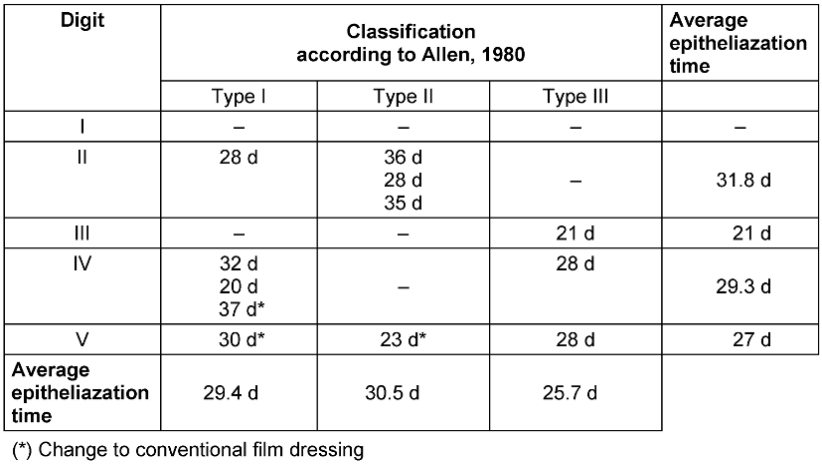
Epithelialization times according to Allen classification of finger cap occlusions

**Table 3:**
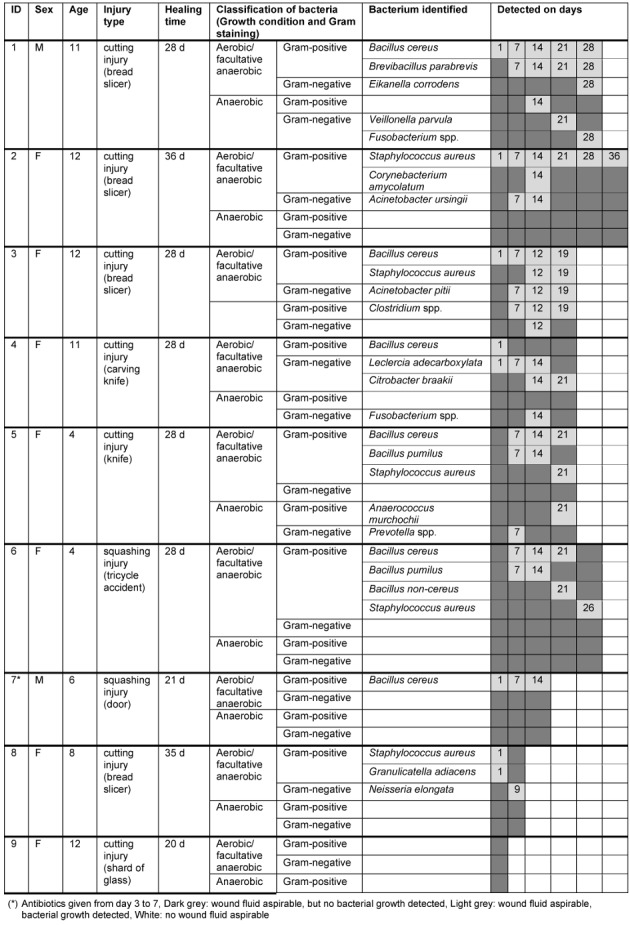
Overview of the bacteria detected in the wound fluid samples aspirated from the silicone finger cap

**Table 4:**
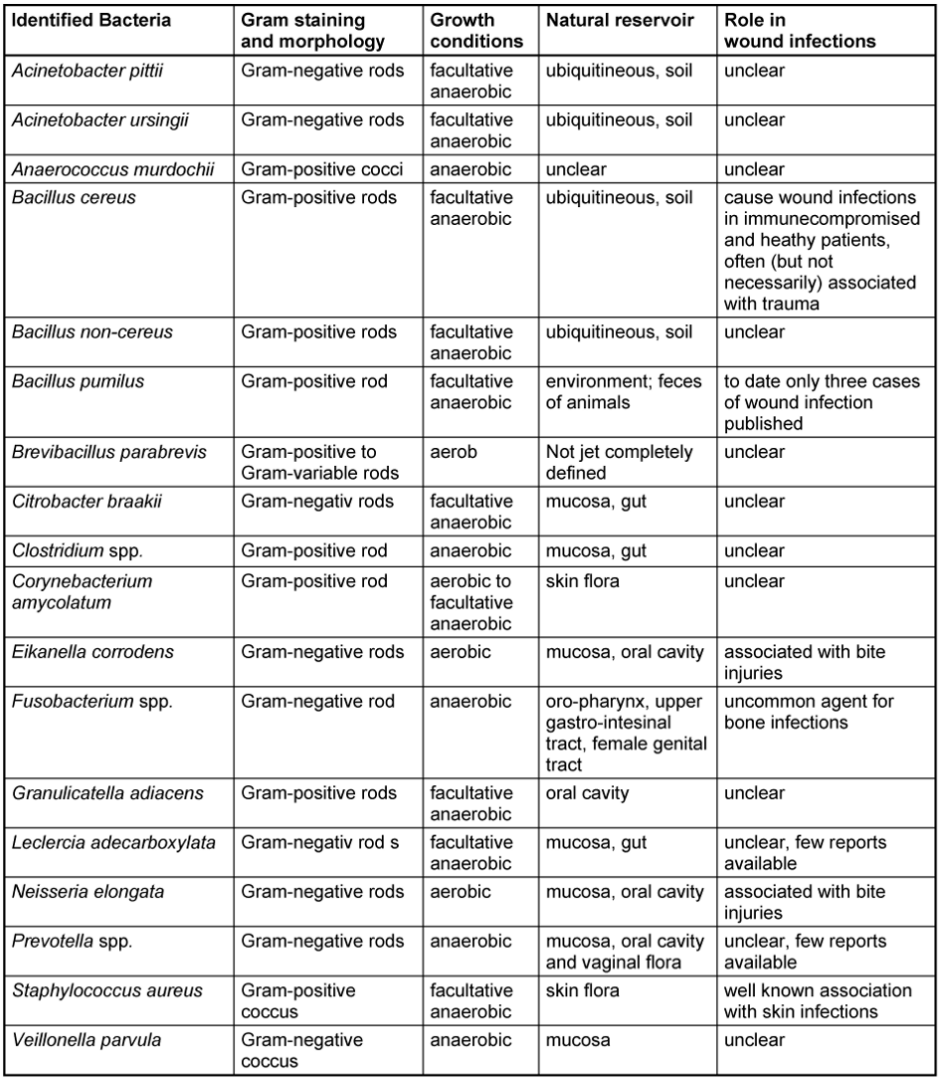
Summary of bacteria identified in the wound fluids
